# Microemulsions: Breakthrough Electrolytes for Redox Flow Batteries

**DOI:** 10.3389/fchem.2022.831200

**Published:** 2022-03-03

**Authors:** Brian A. Barth, Adam Imel, K. McKensie Nelms, Gabriel A. Goenaga, Thomas Zawodzinski

**Affiliations:** ^1^ Department of Chemical and Biomolecular Engineering, University of Tennessee, Knoxville, TN, United States; ^2^ Oak Ridge National Laboratory, Oak Ridge, TN, United States

**Keywords:** microemulsions, redox flow batteries, electrolytes, energy storage, electrochemical devices

## Abstract

Aqueous and non-aqueous redox flow batteries (RFBs) have limited energy and current densities, respectively, due to the nature of the electrolytes. New approaches to electrolyte design are needed to improve the performance of RFBs. In this work, we combined a highly conductive aqueous phase and an organic redox-active phase in a microemulsion to formulate a novel RFB electrolyte. As a proof-of-concept, we demonstrate an RFB using this microemulsion electrolyte with maximum current density of 17.5 mA·cm^−2^ with a 0.19 M posolyte and 0.09 M negolyte at a flow rate of only ∼2.5 ml·min^−1^, comparable to early vanadium electrolyte RFBs at similar flow rates on a per molar basis. The novel active negolyte component is an inexpensive oil-soluble vitamin (K_3_). By combining aqueous and organic phases, the solvent potential window and energy density may be increased without sacrificing current density and new redox couples may be accessed. Microemulsion electrolytes show great promise for improved performance and increased energy densities in aqueous RFBs but the path forward is complex. We end with discussion of areas that need work to achieve the potential of these electrolytes.

## Introduction

Redox flow batteries (RFBs) are a promising energy storage technology. However, both aqueous and non-aqueous RFBs suffer from limitations imposed by electrolyte choice (Luo et al., 2019a). Non-aqueous electrolytes often have low redox species concentrations and poor conductivity, which limits current density (Sun et al., 2017). Aqueous electrolytes have limited energy densities due to the narrow potential window of water, and suitable redox materials are restricted to metal ions or substituted organic redox active species. While progress has been made in the design and implementation of aqueous organic RFBs (Hu et al., 2017; Luo et al., 2019b), an alternative approach to is combine redox-containing oil phases with aqueous electrolyte phases in the same solution.

As part of our work on ‘Breakthrough Electrolytes for Energy Storage’, we adopt this path to broadening the possibilities for RFBs. Microemulsions provide a novel electrolyte design that may overcome existing limitations by providing independent pathways for different functions—ion conduction and redox reaction—allowing us to carry out reactions on redox couples that are not water soluble with the benefit of solution conductivity characteristic of an aqueous system. Microemulsions are spontaneously forming, thermodynamically stable mixtures of oil, water, and emulsifiers, with complex and dynamic nanometer-scale structures. These structures can vary greatly from discrete droplets to bicontinuous networks, where both oil and water are continuous over longer length-scales. There is an expansive history of microemulsion media used in electroanalytical and electro-synthetic studies (Mackay 1994; Rusling 1994; Qutubuddin 1999), but microemulsions have only recently been proposed as RFB electrolytes (Peng et al., 2020; Shen et al., 2021; Peng et al., 2021a; Peng et al., 2021b). We note explicitly that the presence of an electroactive component in the oil phase is the critical aspect of this work, providing access to not only solubility but likely additional reactivity patterns. We also note that the electrochemical conversion may result in the transfer of the oxidized or reduced species from oil to water phase (vide infra) (Mackay 1994), analogous to probe partitioning in aqueous micelle solutions (Oshawa et al., 1980). Nonetheless, we suggest that the microemulsion electrolyte must have one or more active species in the oil-phase if the system is to have meaning outside that of a simple surfactant-containing electrochemical system. This study is intended as a starting point for more extensive work—a proof of concept. Accordingly, we did not seek to optimize conditions or materials but rather simply used what we have used in more fundamental studies. We used methods that we have previously used and, indeed, help standardize in the context of flow batteries. Since we are embarking on a rather different direction for flow batteries, we necessarily build primarily on our recent publications on electrochemistry and structure in microemulsions, but we also want to clearly acknowledge the extensive work in the past.

Electroanalytical microemulsion studies have commonly chosen ferrocene as a model redox species (Iwunze et al., 1990; Mackay et al., 1990; Jha et al., 1995; Mackay et al., 1996; Zu and Rusling 1997; Mo and Li 2007) due to its reversible, outer-sphere electron transfer character (McCreery 2008). We used ferrocene and polysorbate (TWEEN ^®^) 20, a non-ionic surfactant known to be relatively insensitive to solution ionic strength, to probe microemulsion structure-electrochemical behavior relationships (Peng et al., 2020). Unlike previous studies where redox material was added at low concentrations to function as a probe (Iwunze et al., 1990; Mackay et al., 1990; Jha et al., 1995; Mackay et al., 1996; Zu and Rusling 1997; Mo and Li 2007), we suggested that microemulsions could be suitable RFB electrolytes if redox species concentration was increased (Peng et al., 2020). Using microemulsions, it is possible to increase energy density with respect to aqueous RFBs by widening the potential window through surfactant-electrode interactions (Peng et al., 2020; Hou et al., 2017), or the volumetric capacity, through use of organic redox species which undergo multi-electron transfer reactions. It is also possible to increase the achievable current density, relative to non-aqueous RFBs, by increasing the solution conductivity and the concentration of redox active material. The intimate mixing of oil, water, and emulsifier in microemulsion solutions provides a substantial benefit: the choice of organic solvent is no longer constrained by supporting electrolyte solubility because solution conductivity is maintained through the aqueous phase. Therefore, organic solvents and redox species may be chosen with the intent of maximizing redox species solubility.

The ferrocene-polysorbate 20 microemulsion system (Peng et al., 2020) does not have as large of a volumetric capacity or as positive of a redox potential as the more mature vanadium RFB posolyte. However, as a model system it is a viable posolyte for early development of a microemulsion RFB, given its well-documented structure and electrochemical behavior (Peng et al., 2020; Shen et al., 2021). We chose menadione, a vitamin quinone (vitamin K_3_), as a suitable negolyte because of its low cost, lack of toxicity, solubility in toluene, and the ability to undergo two electron transfer reactions. Hence, sacrifices in volumetric capacity originating from solubilizing redox material in only one phase of a multi-phase solution can be partly mitigated. Here we present the first microemulsion electrolyte RFB, identify the strengths and weaknesses of using microemulsion electrolytes, and provide a path for future implementation of this technology.

## Methods

### Microemulsion Preparation

**FIGURE 1:**
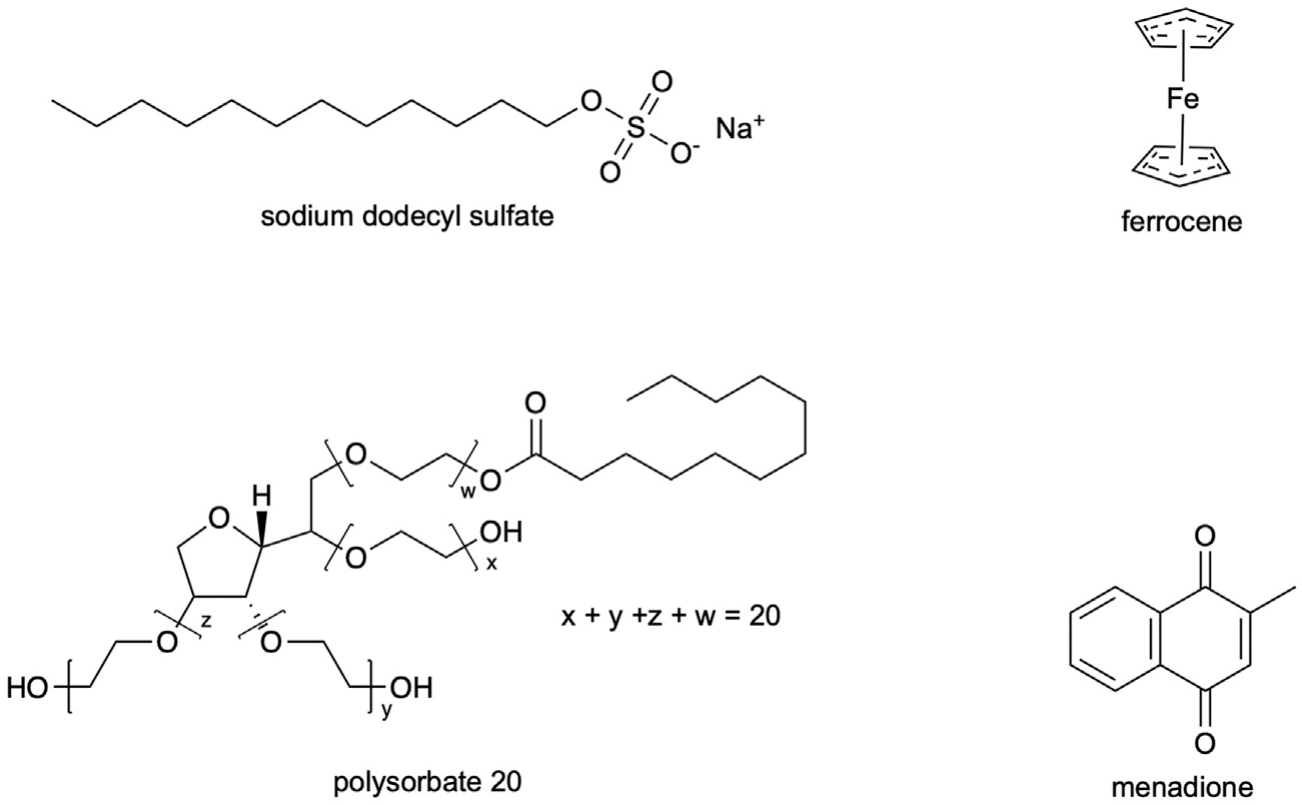
Chemical structures of surfactants and electroactive species.

Polysorbate 20 (Acros, >95%), sodium dodecyl sulfate (SDS; Fisher, ≥ 99%), 1-butanol (Fisher, 99.98%), toluene (Fisher, 99.9%), potassium nitrate (Fisher, ≤ 3ppm heavy metals, iron), ferrocene (Alfa Aesar, 99%), and menadione (MP Biomedicals, ≥ 98%) were used as received. Chemical structures of surfactants and electroactive species are shown in Figure 1.

#### Polysorbate 20 Microemulsions

Emulsifier stock solution was prepared using a 4.7:1 mass ratio solution of polysorbate 20:1-butanol. Electroactive material (ferrocene or menadione) was solubilized in toluene, and then combined with the emulsifier stock solution and KNO_3_ (aq) to reach the desired composition. The resulting mixture was shaken by hand to produce a clear, single-phase microemulsion.

#### Sodium Dodecyl Sulfate Microemulsions

An aqueous emulsifier stock solution was prepared by combining 0.5*m* KNO_3_ (aq), SDS, and 1-butanol in a mass ratio 75/12.5/12.5 (0.5*m* KNO_3_/SDS/1-butanol). Electroactive material (ferrocene or menadione) was solubilized in toluene, and then combined with the aqueous emulsifier stock solution to reach desired composition. The mixture was shaken by hand to produce a clear, single-phase microemulsion.

### Nuclear Magnetic Resonance Spectroscopy

Microemulsion bulk morphology (oil droplets, water droplets, or bicontinuous) was determined from oil and water diffusivities obtained by stimulated pulse echo NMR using a Bruker 400 MHz Ascend spectrometer. Samples were prepared as described above, with the exception that the aqueous salt solution was 10% D_2_O (for signal locking). The applied pulse sequence was Δ = 50 ms, δ = 2 ms, and g varied linearly from 0.963 G/cm to 45.743 G/cm (for microemulsions without redox species present) or 47.187 G/cm (for microemulsions with redox species present). Diffusivities were calculated by fitting the decaying signal areas to the Stejskal-Tanner equation (Stejskal and Tanner 1965):

$$\frac{I}{I_{0}}=e^{-D\gamma^{2}g^{2}\delta^{2}\left(\text{Δ}-\delta /3\right)}$$

where I/I_0_ is ratio of signal intensity to maximum signal intensity (in the absence of an applied z-gradient), D (cm^2^/s) is the diffusivity, γ (rad/(s·G)) is the gyromagnetic ratio of the nucleus, g (G/cm) is the gradient strength, δ (s) is pulse time, and ∆ (s) is the diffusion time.

### Cyclic Voltammetry

Cyclic voltammetry measurements were performed using a BioLogic VMP3 potentiostat and EC-lab software. Approximately 10 ml of microemulsion solution was added to a clean electrochemical cell fitted with a Teflon cap. A glassy carbon working electrode was polished using 5- and 0.05-micron alumina powder slurries on polishing pads prior to use. A glassy carbon working electrode, a saturated calomel reference electrode, and a Pt wire counter electrode were inserted into the solution through the fitted cap. The solution was purged with nitrogen for 10 min prior to data collection and a nitrogen blanket was maintained, without agitating the solution, during data acquisition. Solution resistance was approximated as the high frequency resistance (HFR) obtained by potentiostatic electrochemical impedance spectroscopy (PEIS). Cyclic voltammograms were recorded with iR-compensation using the previously determined solution resistance values.

### Conductivity

**FIGURE 2:**
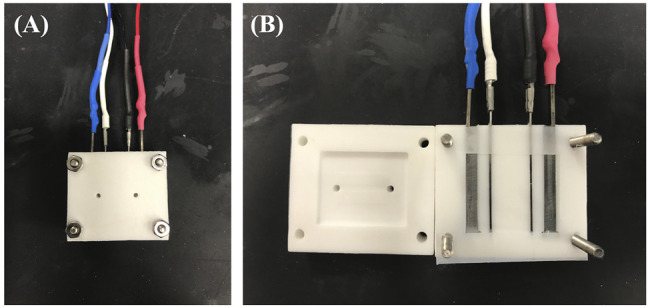
Conductivity cell from **(A)** closed and **(B)** open viewpoint. Outer terminals (blue and red) are power leads and inner terminals (white and black) are sensing leads.

Conductivity measurements were performed using an in-house conductivity cell (Figure 2) operated by a BioLogic SP-200 potentiostat and EC-lab software. Potentiostatic electrochemical impedance spectroscopy was performed by applying 0V DC bias, with a 10mV AC amplitude perturbation while scanning the frequencies from 200 to 1 kHz. The solution resistance was obtained from the HFR intercept determined from the Nyquist plot. Solution conductivity was calculated using

$$\sigma \left(S\cdot cm^{-1}\right)=\frac{1}{HFR}\cdot \frac{length}{cross-sectional\;area}$$

where the length and cross-sectional area are determined by the cell geometry. The methodology and cell were validated by comparison with a conductivity standard solution (Mettler Toledo, 0.00056M KCl), where the measured conductivity was within 2.4% of the standard value.

### RFB Testing

We note at the outset that this study is intended as a starting point for more extensive work—a proof of concept. Accordingly, we did not seek to optimize conditions or materials but rather simply used what we have used in more fundamental studies. We used methods that we have previously used and, indeed, help standardize in the context of flow batteries.

#### Cell Design and Instrumentation

**FIGURE 3:**
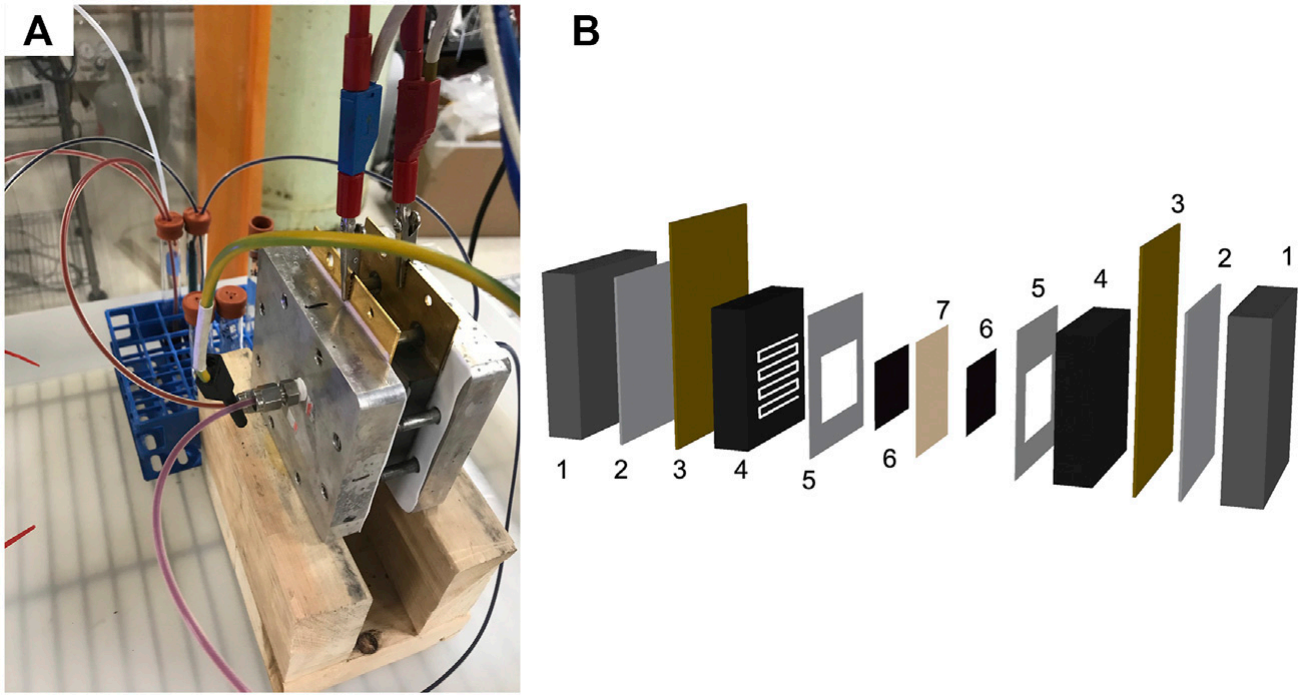
Redox flow battery **(A)** photo and **(B)** exploded diagram of the cell. The cell components are (1) metal plates, (2) polytetrafluoroethylene (PTFE) insulating layers, (3) gold coated current collectors, (4) poco graphite bipolar plates with serpentine flow fields, (5) PTFE gaskets, (6) porous carbon electrodes, and (7) a membrane.

The RFB (Figure 3) consisted of a two-electrode adapted fuel cell hardware design (Aaron et al., 2012), porous carbon electrodes, as received (SGL carbon felt GFD 2.5 mm or SGL Sigracell carbon fiber 350 μm), a Fumatech F-930-P cation exchange membrane, and a MasterFlex peristaltic pump. The battery was operated using a Biologic VSP potentiostat with VMP3B-20 (20 amp) booster controlled by EC-lab software.

#### Polarization Curve Analysis

The RFB was initially charged using chronoamperometry (CA) with an applied voltage of 1.4V, to ∼50% state of charge (SOC). A single-pass flow design was then used for polarization curve analysis to ensure a constant state of charge for electrolytes entering the cell. Due to the large volumes of electrolytes required for single-pass experiments, a relatively slow flow rate of ∼2.5 ml min^−1^ was employed for feasibility. Chronoamperometry was used to determine the steady state current at successive overpotentials starting from the open circuit voltage and incrementing in 25–50 mV steps. The potential was applied for a long enough time to reach the steady state current at each step; 2 min in this study. Electrolyte flow generated by the peristaltic pump caused oscillations in the measured current, and therefore, the current was averaged over a few oscillations at the end of each 2 min potential step to obtain a value for the steady state current. Additionally, PEIS was used to determine the resistance through the cell, following the 2 min potential step. This was performed by superimposing a sinusoidal potential to the existing applied potential for each step. This perturbation had a magnitude of 5–10mV and a frequency of 10–1 kHz. The areal specific resistance was calculated from the product of the cell resistance (obtained from the HFR) and the geometric area of the electrode as shown in the following equation:

$$ASR\;\left(\text{Ω}\cdot cm^{2}\right)=HFR\cdot area$$

#### Preliminary Cell and Electrolyte Optimization

Preliminary cell optimization was performed by modifying the cell architecture (electrode compression) and components (electrodes) and electrolyte compositions. Following modification, the maximum steady state current and areal specific resistance were recorded. Charging was performed by chronoamperometry, as previously described. However, instead of recording the entire polarization curve, only the steady state current and resistance corresponding to a potential step with the largest magnitude overpotential were recorded (E_applied_ = 0.1V).

#### Cycling

A recirculating flow design using a flow rate of 10 ml min^−1^, with 350 μm SGL carbon fiber electrodes, and 30% expansion on the electrodes were used for cycling experiments. The posolyte and negolyte were ferrocene- and menadione-SDS microemulsions with a composition of 8.75% SDS 8.75% 1-butanol, 52.5% 0.5 m KNO_3_ (aq), and 30% redox active species/toluene solution, by mass (4.8 Ah·L^−1^). Galvanostatic cycling was performed at 5 mA cm^−2^, for four cycles.

#### Metrics Calculations

Coulombic efficiency (η_C_), voltage efficiency (η_U_), and energy efficiency (η_E_) were calculated from cycling data using the following equations:

$$\eta_{C}=\frac{Q_{D}}{Q_{C}}$$

$$\eta_{U}=\frac{U_{D}}{U_{C}}$$

$$\eta_{E}=\eta_{C}\cdot \eta_{U}$$

where Q is charge, U is voltage, and the subscripts D and C define discharging and charging steps, respectively.

## Results and Discussion

**SCHEME 1:**
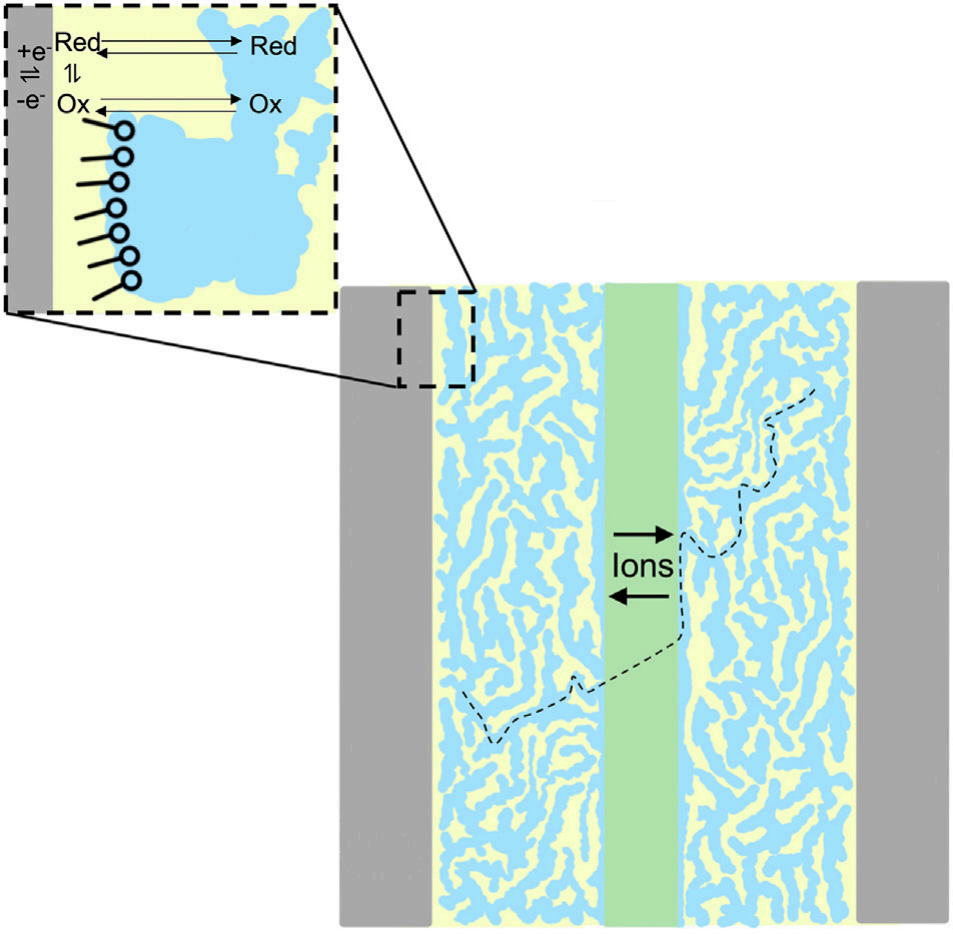
An illustration of a redox flow battery with bicontinuous microemulsion electrolytes comprised of water (blue) and oil (yellow) phases separated by surfactant (inset). Ion conduction (black dashed line) is depicted through the aqueous phase and membrane. Redox reactions are shown in the oil phase (inset).

**FIGURE 4:**
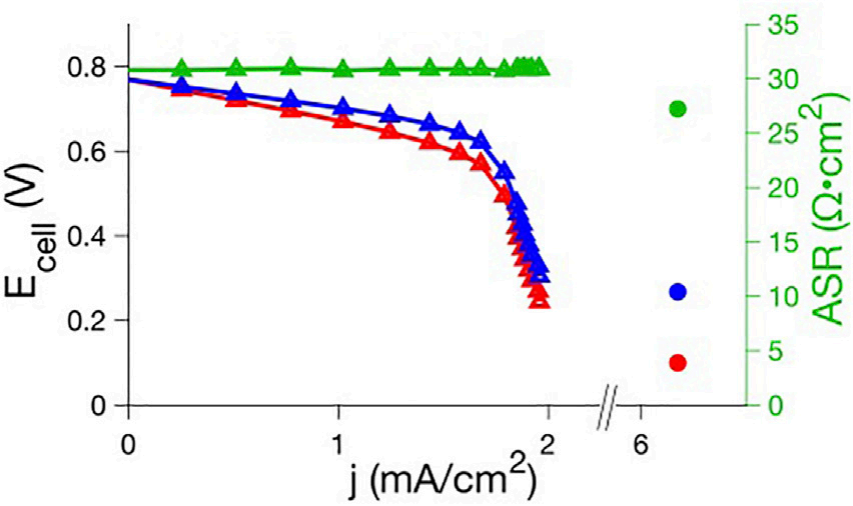
Raw (red) and iR-compensated (blue) polarization curve and ASR (green) data for ferrocene posolyte-menadione negolyte polysorbate 20 microemulsion RFBs. A full polarization curve for a preliminary system (triangles; 89.9% 0.5 m KNO_3_ aq, 7.5% polysorbate 20, 1.6% 1-butanol, 1.0% toluene; carbon felt electrodes with 99% compression; 4.9 mM ferrocene, 2.5 mM menadione) is compared to the maximum current density and ASR for an optimized system (circles; 20% 1.5 m KNO_3_ aq, 47% polysorbate 20, 10% 1-butanol, 23% toluene; carbon fiber electrodes with 30% expansion; 160 mM ferrocene, 80 mM menadione)).

As we previously described (Aaron et al., 2011), polarization curve analysis readily reveals the mechanisms responsible for inefficiencies. Polarization curves provide a correlation between RFB efficiency and the physical behavior (electron transfer rates, mass transport, through-plane membrane resistivity) of electrolytes. Therefore, this analysis can diagnose properties of novel electrolytes that must be altered to optimize performance. We show in Scheme 1 an illustration of a novel microemulsion RFB indicating simultaneous ion conduction through the aqueous phase and redox reactions in the oil phase. The performance of a polysorbate 20 microemulsion RFB at constant ∼50% state of charge, with a 4.9 mM ferrocene posolyte and 2.5 mM menadione negolyte was evaluated using polarization curve analysis, as shown in Figure 4. The RFB used for that experiment consisted of a zero-gap cell (Aaron et al., 2012) Fumasep F-930 cation exchange membrane (∼30 μm thickness) with SGL GFD carbon felt electrodes (5 cm^2^ area, 2.5 mm thickness, 99% compression) where electrolytes passed through the cell at a flow rate of 2.4 ml·min^−1^. There is minimal loss in the kinetic polarization region, indicating facile electron transfer. Ohmic polarization is quantified using the areal specific resistance (ASR), the product of the high frequency resistance and the geometric electrode area. Throughout the porous electrode, ionic and electronic paths exist in parallel, with the electronic path having far less resistance. The ASR is then the sum of electrical resistances in the cell components and the ionic resistance through the membrane which separates the electrodes. Electrical resistances are significantly smaller than ionic resistance through the membrane, and therefore, the ASR is mostly attributed to ionic resistance through the membrane. That is, for an ASR measurement, the electrode behaves as an electronic short circumventing the ionic pathway and the ASR does not probe the resistance of the electrolyte within the electrode. The ASR was ∼30 Ω cm^2^, one to two orders of magnitude larger than the ASR reported for vanadium redox flow batteries (Aaron et al., 2011), indicating significant ionic resistance through the membrane for polysorbate 20 microemulsions.

Following iR-compensation, the polarization curve in the linear region still exhibits a significant negative slope which suggests effects related to mass transport limitations inside the porous electrodes. In this region of the polarization curve, losses associated with transport of ions and redox species through the porous electrode, i.e. distributed resistance effects, dominate (Aaron et al., 2011). To access layers deeper within the electrode, one accrues more and more ionic resistance to bring ions (or charged reactants) in or out of the electrode. Put somewhat differently, functionally, in the polarization experiment, one is forced to bring ions/reagents in or out through the ionic conduction pathway in the porous electrode and thus, after iR correction, this looks like an additional iR loss (which we also refer to as a ‘pseudo-IR) since the polarization curve is linear. This is an inherent *transport* loss that is distinct from that leading to a limiting current.

This loss is further supported by previous studies that have shown the diffusion of ferrocene in this microemulsion system is relatively slow (∼10^–7^ cm^2^ s^−1^) based on electrochemical measurements (Peng et al., 2020). There is also a clear mass transport polarization region associated with reactant starvation causing a maximum current density of ∼2 mA·cm^−2^ (Figure 4). In the absence of this limiting current, the linearly projected current density would be approximately 10 mA·cm^−2^. Total mass transport polarization likely originates from (1) over-compressed electrodes, (2) slow flow rate, (3) low concentration of active material, and/or (4) slow diffusion through the porous electrode.

In an open system such as a flow battery, an important criterion for practical success is a maximized current density. Even with mass transport limitations, normalizing current density to concentration gives ∼400 mA·cm^−2^M^−1^ (j_*max*_ ≈ 2  mA·cm^−2^ for a 5 mM ferrocene solution). In comparison, early vanadium redox flow battery polarization studies reported ∼100 mA·cm^−2^M^−1^ limiting current densities for a similar (2.0 ml·min^−1^) flow rate (Aaron et al., 2011). On a per molar basis, this appears promising. However, application requires larger total current densities which can be optimized through cell (electrode type, electrode compression) and electrolyte (chemistry and concentrations of salt, oil, surfactant, cosurfactant, redox species) engineering. Preliminary cell optimization was performed using polysorbate-based microemulsions. Electrode type, electrode compression, and microemulsion composition were varied in different combinations and the maximum current density was recorded. The best performance obtained from polysorbate 20 microemulsion electrolytes was a maximum current density of ∼6 mA·cm^−2^ for a similar state of charge and flow rate using a 160 mM ferrocene posolyte and an 80 mM menadione negolyte. Better performance was achieved through cell and electrolyte modifications by replacing carbon felt electrodes with thinner carbon fiber electrodes (SGL carbon fiber electrode with 5 cm^2^ area and 350 μm thickness), decreasing the electrode compression (-30% “compression”) and changing redox species concentration and microemulsion composition. Despite the changes, the maximum current increased by only a factor of ∼3 (Figure 4). In the absence of a full polarization curve, it is unclear how ionic or mass transport resistances contribute to the total loss. However, it is unlikely this polysorbate 20 electrolyte will be of practical use given the low current density and the large amount of surfactant needed to form a microemulsion, which limits energy density as well. Surfactant and cosurfactant are used only to maintain a stable microemulsion, and highly efficient surfactant/cosurfactant combinations should be used. Surfactant efficiency refers to the amount of oil and water that can be co-solubilized per amount of surfactant and is a common metric when evaluating (and optimizing) surfactants for microemulsion formulation (Salager et al., 2005). Polysorbate 20/1-butanol has poor efficiency with respect to toluene in this system: 37% polysorbate 20 and 8% 1-butanol is required to reach 30% toluene solutions. This microemulsion was chosen as an initial system based on our previous characterization of the phase behavior and corresponding electrochemistry. There is a complex relationship between microemulsion composition, nanometer domain structure, viscosity, conductivity, diffusion, and electrochemical behavior including redox potential and electron transfer kinetics (Mackay et al., 1990; Mackay et al.,1996; Peng et al., 2020; Shen et al., 2021). Therefore, prior knowledge of composition-property relationships could aid in analysis for electrolytes that had not yet been tested in RFBs. The polysorbate 20 system used in this study is bicontinuous (Peng et al., 2020), having both connected oil and water domains. Bicontinuous microemulsions are desirable because oil concentration is maximized while maintaining connected aqueous domains, which are needed for a conductive solution. Additionally, diffusion of oil-soluble redox active species will be greater than in oil droplet systems, increasing current density. However, polarization curve data shows that even when redox species concentration is near maximal for this system, the current density is only ∼6 mA cm^−2^ (under the given experimental conditions). Due to the limitations of the polysorbate system, a microemulsion with a more efficient surfactant (SDS) was then tested, to determine the effect of changing surfactant on polarization curve behavior and current density. Polarization analysis with SDS microemulsions was performed using the cell architecture (electrode type and compression) for which the polysorbate microemulsion system exhibited maximum current density.

**FIGURE 5:**
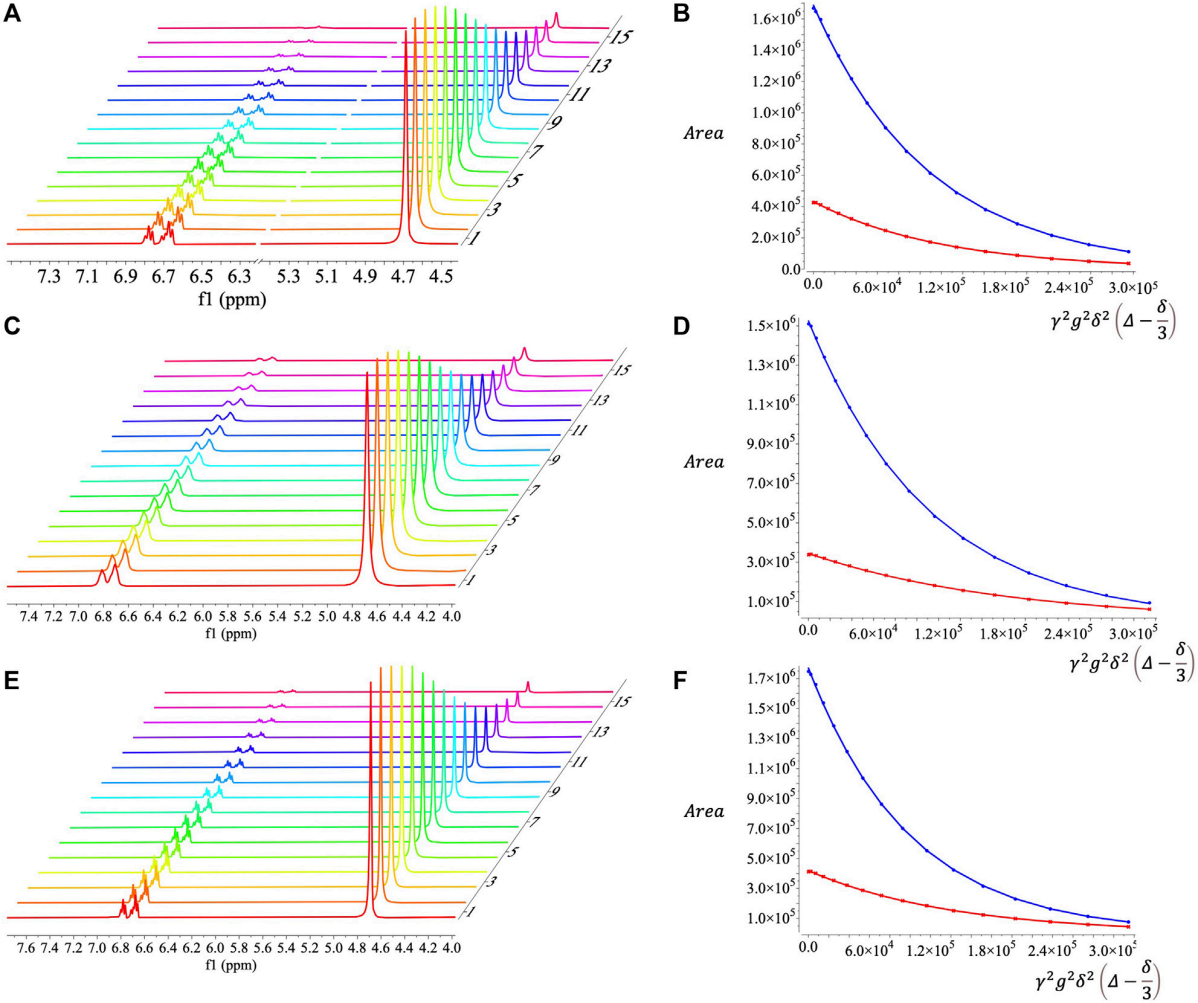
Diffusion NMR spectra and fits for decaying signal peak area as a function of gradient strength for **(A, B)** SDS microemulsions, **(C, D)** SDS microemulsions with ferrocene, and **(E, F)** SDS microemulsions with menadione. Red and blue curves in **(B, D, F)** correspond to peak areas for protons in toluene and water, respectively.

SDS is a well-characterized surfactant (Chevalier and Zemb 1990; Ben-Shaul and Gelbart 1994; Acosta et al., 2003; Acosta et al. 2008) that has been used in electrochemical studies (Georges et al., 1987; Abbott et al., 1992; Mackay et al., 1996). Microemulsions up to 30% toluene can be formed using only 8.75% SDS, 8.75% 1-butanol, and 52.5% 0.5 m KNO_3_ aq., by mass. These SDS microemulsions were verified to be bicontinuous using proton diffusion NMR (Figure 5A). The decays in peak areas corresponding to protons from water and toluene were fit to decaying exponential functions (Figure 5B) to obtain diffusivities (Table 1). Errors in the fits (Table 1) were several orders of magnitude smaller than the determined diffusivities, which are reasonable in magnitude. The diffusivities of toluene and water are on the same order of magnitude and therefore the microemulsion is considered to be bicontinuous (Lindman et al., 1999). The idea behind this conclusion is that for a non-continuous oil or water phase, NMR diffusion measurements will report orders of magnitude differences since the non-continuous phase. Diffusion NMR spectra for SDS microemulsion solubilized ferrocene (Figure 5C) and menadione (Figure 5E) exhibited similar signal decays (Figures 5D,F, respectively) and diffusivities (Table 1) to the blank microemulsion. The small changes in water and toluene diffusivities between blank and redox solubilized systems may result from minor changes in interfacial curvature of the surfactant layer or from solvation effects (Lindman et al.,1999).

**TABLE 1:** NMR diffusivities for SDS microemulsions.

Microemulsion	Toluene (x 10^−6^ cm^2^ s^−1^)	Toluene fitting error (x 10^6^)	Water (x 10^−6^ cm^2^ s^−1^)	Water fitting error (x 10^6^)
Blank	8.342	0.0347	9.211	0.02915
Ferrocene	5.509	0.02693	8.996	0.02344
Menadione	7.072	0.03078	10.00	0.03358

**FIGURE 6:**
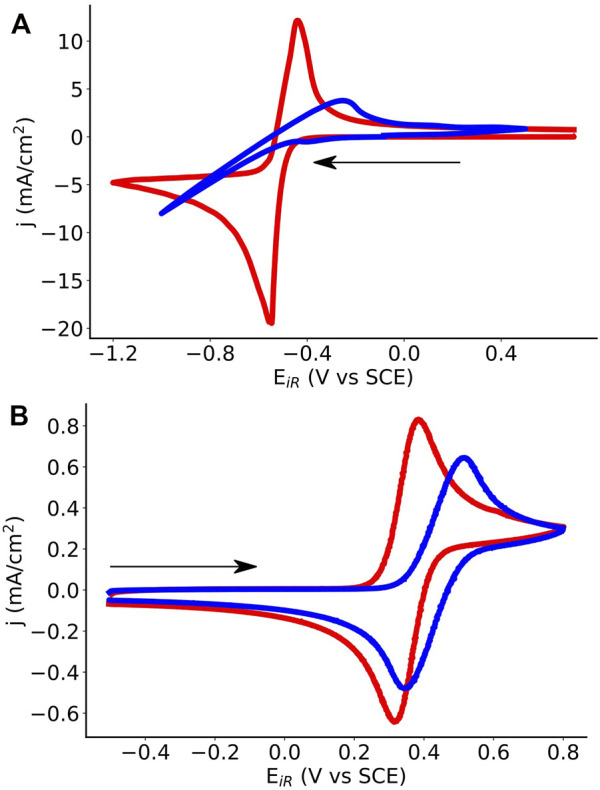
Cyclic voltammograms comparing SDS (red) and polysorbate 20 (blue) microemulsions for **(A)** menadione negolyte and **(B)** ferrocene posolyte solutions. SDS microemulsions are 54% 0.5 m KNO_3_ (aq), 28% toluene, 9% SDS, and 9% 1-butanol, by mass. Polysorbate microemulsions are 22% 2 m KNO_3_ (aq), 31% toluene, 39% polysorbate 20, and 8% 1-butanol, by mass. Menadione concentrations are 0.13 M in SDS and 0.3 M in polysorbate microemulsions. Ferrocene concentrations are ∼5 mM in SDS and polysorbate microemulsions. Voltammograms were recorded using glassy carbon working electrodes, saturated calomel reference electrodes, and Pt wire counter electrodes using a scan rate of 50 mV·s^−1^. Sweep direction is indicated by the arrow.

The effect of surfactant choice on electrochemical behavior of ferrocene and menadione was qualitatively analyzed using cyclic voltammetry (CV). Electron transfer kinetics are more facile for both menadione (Figure 6A) and ferrocene (Figure 6B) in SDS microemulsions compared to polysorbate 20 microemulsions as indicated by the shape of the cyclic voltammogram. Additionally, the peak current density for ferrocene is approximately three times larger for the SDS microemulsion than the polysorbate 20 microemulsion. This behavior may be explained by both faster kinetics and increased diffusion. Menadione electron transfer in a polysorbate 20 microemulsion (at ∼30% toluene) appears irreversible, but in an SDS microemulsion the voltammogram exhibits both oxidation and reduction peaks. While the effect of microemulsion component ratios on electrochemical behavior was previously investigated (Peng et al., 2020), these findings emphasize the importance of surfactant chemistry.

**FIGURE 7:**
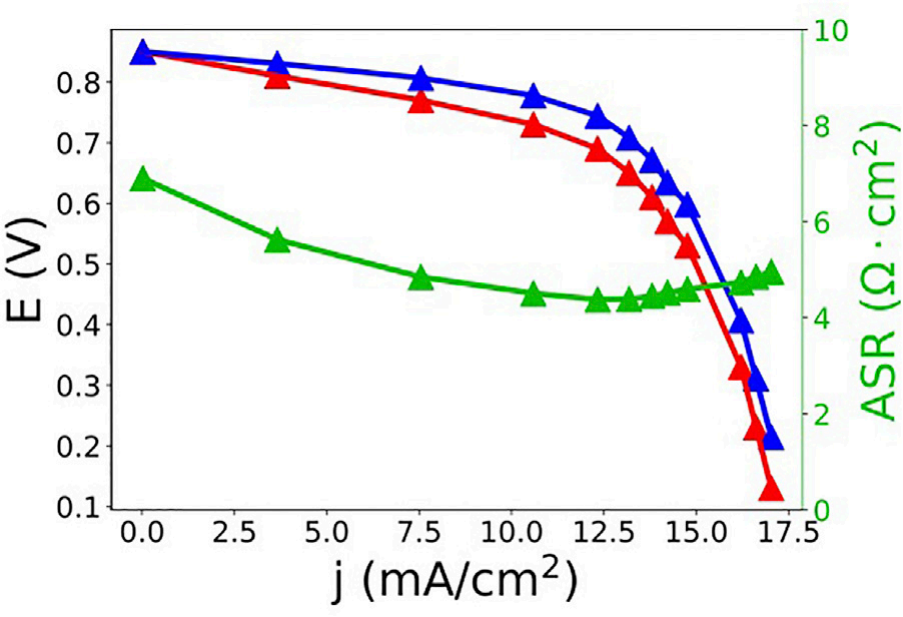
Raw (red) and iR-compensated (blue) polarization curve and ASR (green) data for a ferrocene (192 mM) - menadione (89 mM) SDS microemulsion (28% toluene, 9% SDS, 9% 1-butanol, 54% KNO_3_ aq., by mass) RFB.

How does RFB performance using SDS microemulsions compare to polysorbate 20 microemulsions? A polarization curve for a ferrocene (192 mM) - menadione (89 mM) SDS microemulsion (28% toluene, 9% SDS, 9% 1-butanol, 54% KNO_3_ aq., by mass) RFB is shown in Figure 7. Similar to the polysorbate 20 system, mass transport loss both in the form of diffusive transport loss (linear region) and concentration polarization loss (limiting current) is observed. Again, we note that while both effects have an origin in mass transport, they are somewhat distinct. Linear mass transport loss originates from transport of ions through the porous electrode, as described above, while the loss at greater current densities originates from reagent starvation, which is essentially determined by the overall solution flow rate into the cell, the concentration of reagents and intrinsic permeability of the electrode. The presence of these losses is due, in part, to experimental design. The polarization curves were performed using single-pass flow to ensure a constant state of charge during measurement. Due to the single-pass experimental design, a slow flow rate (2–3 ml·min^−1^) was used to reduce the amount of sample required. As flow rate slows, there is likely to be increased mass transport loss throughout the polarization curve (Sun et al., 2014). However, there is a greater pseudo-iR loss, loss originating from ion coupled-redox species mass transport in the porous electrodes, in the polysorbate RFB than the SDS RFB, as demonstrated by the slopes of the iR-compensated polarization curves in the linear region. The SDS iR-compensated polarization curve exhibits a slope that is approximately an order of magnitude smaller than that of the polysorbate system. While this may be as dependent on the carbon electrode as the microemulsion, the exact nature of microemulsion-porous carbon electrode interaction has not been thoroughly investigated yet.

Table 2 compares the maximum current densities, ASRs, and energy densities between polysorbate 20 and SDS microemulsion electrolytes used in an RFB with similar concentrations of ferrocene (∼0.16 M for the polysorbate 20 microemulsions and ∼0.19 M for the SDS microemulsions) and menadione (∼0.08 M for the polysorbate 20 microemulsions and ∼0.09 M for the SDS microemulsions). Maximum current density reached 17.5 mA cm^−2^, three times the mass transport limited current density observed for the polysorbate 20 system using similar redox species concentrations. ASR decreased from 27 to ∼5 Ω cm^2^ when polysorbate was replaced with SDS. The decrease in ASR results from increased ionic transport. Surprisingly, the conductivity of the SDS system (28/9/9/54 toluene/SDS/1-butanol/0.5 m KNO_3_, by mass) was 14 mS·cm^−1^ compared to 21 mS·cm^−1^ for the polysorbate system (1/7.5/1.6/89.9 toluene/polysorbate 20/1-butanol/0.5 m KNO_3_, by mass). However, ASR accounts for membrane resistance and it is currently unclear how different microemulsions interact with porous carbon electrodes and membranes to influence ionic transport. While it is beyond the scope of this work, a thorough understanding of these interactions is needed if microemulsion RFBs are to be optimized.

**TABLE 2:** Microemulsion RFB—best performance comparison.

Microemulsion	Max j (mA·cm^−2^)	ASR (Ω·cm^2^)	C (Ah·L^−1^)	E (Wh·L^−1^)
Polysorbate 20	6.2	27	4.29	0.5
SDS	17.5	∼5	4.82	0.5
Theoretical best	*17,700	*0.13	54	124

The observed decrease in resistance most likely contributed to the increase in current density as mass transport losses in the electrode lead to uncompensated losses at mid-range current densities.

Volumetric capacities and energy densities are similar for both systems because the total amount of toluene solubilized, and therefore total concentration of redox active material was nearly equivalent. A theoretical “optimized” microemulsion electrolyte can be hypothesized from literature values and results from previously mentioned polarization curve studies. Using Ohm’s law and the ASR from the SDS polarization curve experiment (5 Ω cm^2^), a theoretical current density can be determined. For a bicontinuous microemulsion with an extended potential window of ∼2.3 V (Iwunze et al., 1990), the maximum current density would be 460 mA·cm^−2^. However, this is not an optimized ASR value. Our lab has previously demonstrated that current densities in VRFB could be increased to 5 A/cm^2^, in part by reducing the ASR to only 0.13 Ω cm^2^ (Elgammal et al., 2017). This is a dramatic improvement over early VRFBs which reported ASR values of ∼4 Ω cm^2^ (Sun and Skyllas-Kazacos 1992). If the ASR in the microemulsion RFB shown here was reduced to 0.13 Ω cm^2^, the theoretical best current density, in the absence of other voltage losses (polarization, pseudo-iR, and mass-transport), would be 17.7 A/cm^2^, due to the large potential window. Obviously, this extrapolation likely dramatically overstates the achievable increase since other factors besides ASR will undoubtedly dominate the losses as ASR is improved (see the final discussion below). However, decreasing the ASR can have a major effect.

If a nonpolar redox species that undergoes two electron transfer reactions (e.g., quinones) was dissolved in this microemulsion at a concentration of 1 M overall, the theoretical volumetric capacity 54 Ah·L^−1^. If the cell voltage was at the limit of the potential window (2.3 V), the energy density would be 124 Wh·L^−1^. Although, the SDS and polysorbate systems do not yet approach the theoretical best, they provide a proof of concept which can be improved through future optimization, as previously shown for VRBs in our lab (Elgammal et al., 2017). Furthermore, this is more directly comparable to a non-aqueous redox flow battery (NARFB) and our *nonoptimized* current density without any normalization is already on the same order of magnitude of many NARFBs. Achieving high current density, while not the ONLY limitation of non-aqueous systems, is critical to keeping cell counts and concomitant materials costs low and, indeed, was the primary way in which RFBs advanced in the past decade. In fact, because of the low conductivity of non-aqueous media, current density is quite limited in NARFBs and that is a major *weakness* of such systems.

**FIGURE 8:**
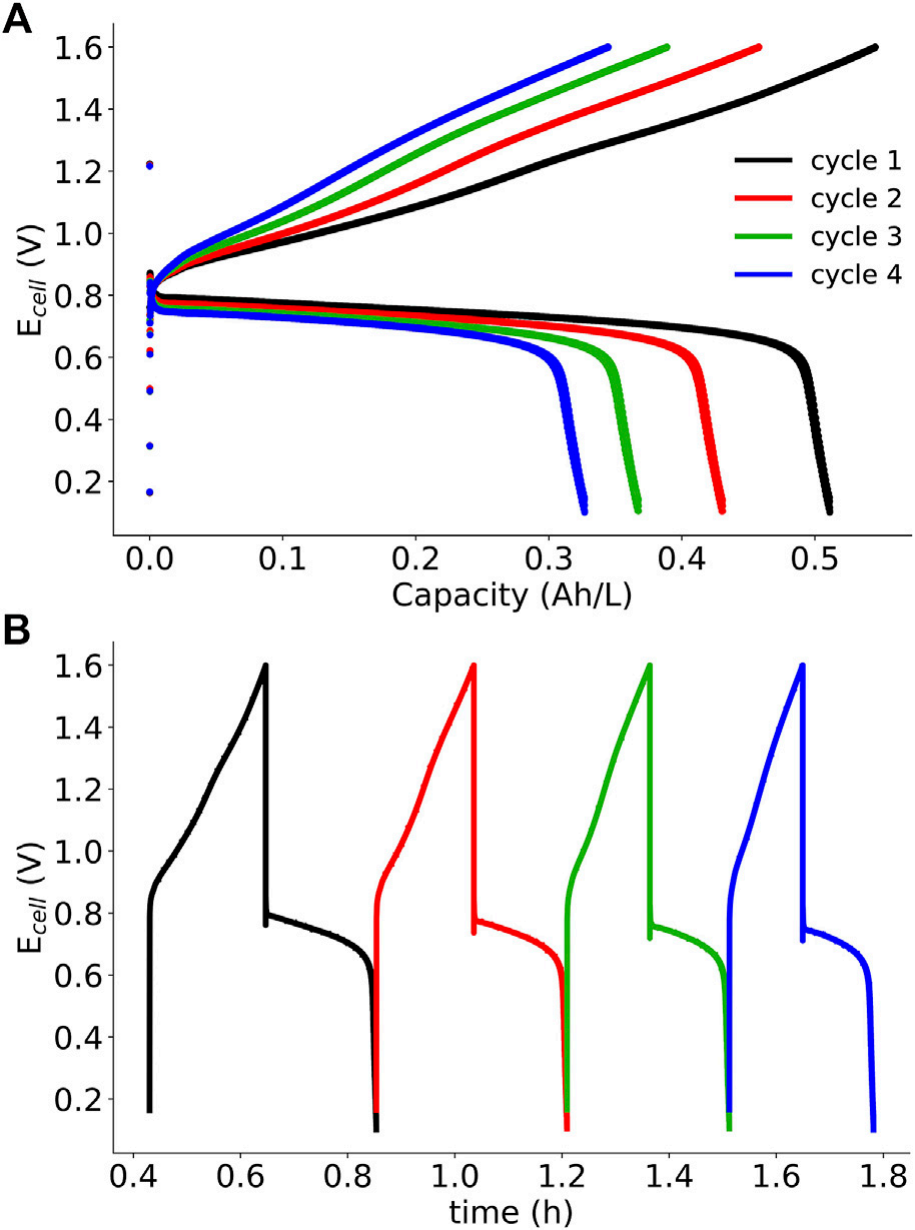
SDS microemulsion RFB galvanostatic cycling data (5 mA·cm^−2^) for four cycles: **(A)** E_cell_ vs capacity and **(B)** E_cell_ vs time. SDS microemulsions are 8.75% SDS 8.75% 1-butanol, 52.5% 0.5 m KNO_3_ (aq), and 30% redox active species/toluene solution. Ferrocene (192 mM) and menadione (89 mM) were the posolyte and negolyte redox active species, respectively.

SDS microemulsions show improved performance over polysorbate 20 microemulsions, but how stable are these electrolytes? To test the stability of the system, galvanostatic cycling was performed at 5 mA cm^−2^. Charge-discharge cycles are shown in Figure 8, and the derived efficiency metrics (Coulombic, voltage, and energy) and capacities per cycle were calculated are presented in Table 3. Over four cycles, Coulombic efficiency (η_C_) was relatively constant (94–95%), but less than 99%, indicating possible crossover or side reactions (Winsberg et al., 2017). Additionally, charge and discharge capacity decreased by at least 35%, another indication of crossover or coupled homogeneous reactions consuming redox active material. Following cycling experiments, the membrane was removed and examined. Material from the electrolyte had been taken up into the membrane, which is another indication of possible crossover. Microemulsion-membrane interactions are complex due to the presence of polar and non-polar components, and these interactions will be further investigated in a future work. It is possible that coupled homogeneous reactions involving menadione are responsible for capacity fade given the limited stability of most quinones (Kwabi et al., 2018). In an aprotic solvent, it is expected that menadione would be reduced to a dianion, which may be reactive. Additionally, microemulsions have been frequently used as microreactors to catalyze reactions by confining reactants to smaller domains (Rusling 1994). The confinement of reactive quinones may amplify coupled homogeneous reaction rates leading to the observed capacity fade.

**TABLE 3:** Microemulsion RFB metrics.

Cycle	η_C_ (%)	η_U_ (%)	η_E_ (%)	Q_D_ (mA·h)	Q_C_ (mA·h)
1	94	60	56	5.1	5.5
2	94	58	54	4.3	4.6
3	95	56	53	3.7	3.9
4	95	55	52	3.3	3.4

Voltage (η_U_) and energy (η_E_) efficiencies range from 55 to 60% and 52–56%, respectively; values which are lower than early vanadium RFBs. Voltage losses are the result of the combined overpotentials: kinetic, ohmic, and mass-transport. Through polarization curve analysis, it was determined that mass-transport losses through the porous electrodes and ohmic losses through the membrane were significant. Although cycling experiments were performed at a greater flow rate than polarization curve experiments (∼10 ml/min compared to ∼2.5 ml/min), mass transport limitations may still be responsible for the observed voltage inefficiencies.

While the demonstrated use of microemulsion based electrolytes provided here is a first step, there is a substantial hill to climb to make these electrolytes as useful as possible. Electrolyte design is motivated by the following objectives: (1) maximize energy density, (2) maximize current density, (3) minimize capacity fade, and (4) minimize cost. Each objective is dependent on microemulsion and redox species choice, as well as RFB cell design and operation parameters. For typical oil-water-surfactant-cosurfactant microemulsions, there are 15 independent variables (for a quaternary mixture, three of the four component fractions are independent, and one is constrained) that must be optimized to meet these objectives (Table 4). A closer look at the first objective, maximizing energy density, will illustrate the complexity of this problem. Energy density can be described mathematically as:

E=C⋅U=nF[redox]⋅U

where E is the energy density (J/L), C is the volumetric capacity (coulomb/L), U is the voltage (V), *n* is the mole of electrons transferred per mole of redox species, F is Faraday’s constant (coulombs/mole electrons), and [redox] is concentration of redox species (M). The simplest case for a microemulsion electrolyte is a nonpolar redox species which exhibits a single, reversible outer-sphere electron transfer (e.g., ferrocene), fixing *n* at one. Microemulsion formulation can increase energy density by increasing redox concentration ([redox]) and/or shifting the redox potential of the half reaction (which will influence the cell voltage, U). Assuming that all surfactant and cosurfactant are at the amphiphilic interface, the redox concentration is determined by solubility in the oil phase. Therefore, it is logical to choose an oil that will maximize redox solubility and a microemulsion composition that will maximize the oil phase. However, for an RFB application, this is achieved under the constraints that the microemulsion is single-phase and conductive.

**TABLE 4:** Optimization objectives and independent variables.

Number of variables	Objectives (dependent Variables)	Independent variables
1	energy density	redox species^a^
2	current density	salt/acid/base^a^
3	capacity fade	oil^a^
4	cost	surfactant^a^
5		cosurfactant^a^
6		temperature^a^
7		[redox]^a^
8		Ionic strength/pH^a^
9		*oil phase fraction* ^a^
10		*aqueous phase fraction* ^a^
11		*surfactant fraction* ^a^
12		*cosurfactant fraction* ^a^
13		flow rate^b^
14		membrane^b^
15		electrode^b^
16		flow field geometry^b^

In an oil-water-surfactant-cosurfactant system, the fraction of one of the components will be constrained (dependent).

a Microemulsion variables.

b RFB, variables.

Not all compositions of an oil, water (or brine), surfactant, cosurfactant mixture will generate single-phase microemulsions; water- or oil-swollen micelles, lyotropic liquid crystals, and macroemulsions are all possible outcomes (Moulik and Paul 1998). If surfactant content is too low, macroemulsions are often formed. Macroemulsions are kinetically stable dispersions of droplets that are several orders of magnitude larger than microemulsion domains. Given sufficient time, macroemulsions will phase separate into a microemulsion layer with excess oil and/or water layers. Energy density will decrease if excess phases are formed which do not participate in electrochemical reactions, and inefficiency will increase if energy input is needed to prevent phase separation. On the contrary, if surfactant content is excessive, energy density will decrease. The only purpose of the surfactant is to reduce the interfacial tension between the oil and water phases, allowing microemulsions to form. Once a single-phase microemulsion forms, additional surfactant will (generally) increase solution volume and thus, reduce energy density. Furthermore, the microemulsion must be conductive for electrochemical applications, implying a continuous aqueous network. Microemulsion conductivity is strongly correlated to structure, and while large oil content favors increasing energy density, it also favors water-in-oil droplet structures which are insulating. Therefore, an upper limit on oil content exists for each system that must not be exceeded. Oil-in-water droplets and bicontinuous microemulsions both satisfy the conductivity requirement, but bicontinuous systems will maximize energy density while maintaining a continuous aqueous phase. This means, for each possible quintenary (redox species, oil, brine, surfactant, cosurfactant) microemulsion, the component- and composition-dependent phase behavior must be understood if bicontinuous systems with minimal surfactant are to be screened. This is a non-trivial, time-consuming task.

Additionally, energy density can be increased through microemulsion formulation by modifying the cell voltage term. This is accomplished by (1) extending the aqueous potential window (Peng et al., 2020) to enable to use of previously inaccessible redox species or (2) shifting redox potentials to increase cell voltage (Mackay et al., 1996). The mechanisms for these behaviors are not yet clear.

The remaining objectives are just as complex and may include RFB cell variables, as well. For example, current density is a function of potential-dependent kinetics for electron transfer at a porous electrode while conductivity is maintained through a membrane and mass transport is a function of flow rate. The electrode- and membrane interactions with the microemulsion, as shown in this work, further complicate microemulsion electrochemistry. Thus, improving the performance, i.e. current density or power density, of an RFB based on microemulsions will entail control over interactions with the physical cell components (i.e. electrodes and membranes). There are significant questions related to the wetting of each of those components and how it is affected by the surfactants, for example. Mass transport and interfacial electron transfer within porous electrodes is a major point of emphasis for achieving high performance. Significant progress is needed to optimize microemulsion formulation, specifically to increase the attainable solubility of the redox active species (to realize energy densities equal to or high than those achieved in other systems), and to understand and control interactions of the various electrolyte constituents with cell components.

Though the matrix of optimizations does indeed appear to be complex, this is a space that our group has navigated before with major gains in performance. Improvements in membranes, electrodes and cell design all contributed to these gains. The use of microemulsions in electrochemical conversions for RFBs and beyond offers a unique variable for manipulating the reaction pathways possible.

## Conclusion

In this work, we have demonstrated the use of microemulsion electrolytes as potential “breakthrough” electrolytes by showing their use in model microemulsion RFBs. Microemulsions can incorporate novel organic redox species which were previously incapable of being used in combination with aqueous systems or suffered from poor performance in non-aqueous systems due to low solubility or poor conductivity. Microemulsion RFB performance was improved by changing electrolyte composition and by hardware modification. Maximum current densities for the optimized microemulsion redox flow battery are comparable to many NARFBs. However, performance was limited by inefficiencies and capacity fade. Voltage and energy efficiencies were less than 60% and most likely arise from mass transport overpotentials, as identified by polarization curve analysis. Coulombic inefficiency and Capacity fade may be related to crossover as well as coupled side reactions involving reactive menadione anions confined to small nonpolar domains. Similar to many quinone RFBs, overcoming capacity fade will lead to increased applicability of the microemulsion RFB. Based on this early work microemulsions have great potential as electrolytes if major efforts aimed at optimizing composition of the electrolytes and tailoring cell components for this application are undertaken. The unique combination of conductive aqueous and reactive oil phases may overcome limitations of existing aqueous and non-aqueous systems, furthering the promise of RFBs as energy storage devices.

## Data Availability

The raw data supporting the conclusions of this article will be made available by the authors, without undue reservation.
